# Flight safety assessment based on a modified human error risk quantification approach

**DOI:** 10.1371/journal.pone.0302511

**Published:** 2024-04-29

**Authors:** Yundong Guo, Xinshi Suo

**Affiliations:** 1 School of Aeronautical Engineering, Nanjing Vocational University of Industry Technology, Nanjing, China; 2 Aeronautic Intelligent Manufacturing and Digital Health Management Technology Engineering Research Center, Nanjing Vocational University of Industry Technology, Nanjing, China; Cyprus International University Faculty of Engineering: Uluslararasi Kibris Universitesi Muhendislik Fakultesi, TURKEY

## Abstract

In risk and safety assessments of aviation systems, engineers generally pay more attention to the risks of hardware or software failure and focus less on the risks caused by human errors. In this paper, a (FRAHE) method is proposed for identifying this critical error type and determining the risk severity of human errors. This method accounts for the human error probability as well as the impacts of human errors on the system. The fuzzy inference approach is employed in this paper to address the uncertainty and issues of imprecision that arise from insufficient information and scarce error data and a risk assessment model of human error is developed. The model can be used to precisely describe the relationship between the output risk severity and the input risk indicators, including the human error probability, the error impact probability, and the human error consequence. A case study of the approach task is presented to demonstrate the availability and reasonability of the model. The risk-based modeling method can not only provide valuable information for reducing the occurrence of critical errors but also be used to conduct prospective analyses to prevent unsafe incidents or aviation accidents.

## 1. Introduction

The continuous development of airborne equipment and automation has greatly improved the reliability and safety of aircraft, but the occurrence of aviation accidents caused by human error have not been significantly decreased. An investigation by the International Civil Aviation Organization (ICAO) indicated that more than 75% of the risk in civil aviation transportation is either directly or indirectly related to human errors [1, 2]. In a large-scale human‒machine system, operators tend to make mistakes due to the influence of the unfriendly context and physical limitations [3–5]. A high operation error probability that has a strongly negative impact on system safety is called a critical human error. To effectively prevent and control serious errors, quantifying the human error risk and further identifying critical human errors is necessary to enhance flight safety.

Numerous human error analysis methods have been proposed for identifying critical human errors. Kirwan [6] reviewed more than 30 human reliability analysis (HRA) techniques and then categorized these approaches, which can be roughly separated into two phases: the first-generation HRA and the second-generation HRA. First-generation HRA was developed mainly based on the task process, where many methods involve variants of a single method [7, 8]. For example, the human error rate prediction technique (THERP), which was developed by the US Nuclear Regulatory Commission, is a variant of the operator action tree (OAT) [9]. The greatest strength of the OAT lies in its division of human response behavior into three stages, i.e., perception, diagnosis and execution. It focuses on determination of the probability of correct diagnosis events, which however does not take the influence of situational factors into account. The main contributions of the THERP are that it provides event tree models, constructs human error basic databases and develops the concept of performance shaping factors (PSFs). Although this technique is a structured engineering analysis method, it lacks uniformity in the application of scenario models and databases. The success likelihood index method (SLIM), human cognitive reliability (HCR), and human error assessment and reduction technique (HEART) were developed under the assumption that humans have inherent defects, such as mechanical or electrical defects [10–12]. Although the SLIM can quantify human reliability for a task when all influencing factors are known, these factors are difficult to obtain in some cases. The strength of the HCR lies in its use of simulator experience data to analyze human cognitive reliability; however, it is difficult to identify which process human decision-making belongs to. The HEART is a powerful tool which provides more than 30 error-producing conditions (EPCs) to compute human reliability. However, it can only be applied to standalone tasks and cannot handle sequential tasks. In the first-generation methods, the analytical task is decomposed into a series of subtasks, and some basic probabilities of human errors are given according to expert discussion. These probabilities are subsequently adjusted by PSFs or EPCs, which represent the context that affects human actions [13]. Second-generation HRA approaches, such as the technique for human error analysis (ATHEANA) [14], the cognitive reliability and error analysis method (CREAM) [15], task analysis for error identification (TAFEI) [16], and the standardized plant analysis risk-human reliability analysis method (SPARH) [17], were developed on the basis of the human cognitive process model. The ATHEANA provides a novel analytical framework by combining human error mechanisms with human error consequences, and it develops the EPC database; however, it is still not publicly available. The CREAM introduces a human information processing model to improve the operator behavior model, but its quantitative analysis is only based on a small amount of human factor data and thus is oversimplified. The TAFEI is the theoretical and empirical development of HRA and is superior to heuristic methods. However, its complicated analysis process greatly hinders its popularization in practice. The SPARH establishes a two-step process to identify nominal human error probability by imposing PSFs upon it. Despite a simplified method, the SPARH has inherent limitations to modeling and analysis. These techniques represent attempts to integrate context factors into human information processing, including observation, interpretation, planning, and execution. All the HRA methods can be utilized to calculate the human error probability and support the technically complex assessment of potential human error risk. However, these studies are overfocused on human reliability quantification and pay insufficient attention to the effect of human errors on the system. This disadvantage imposes restrictions on the application of these human error identification techniques to the risk assessment of human error.

By focusing on the risk and safety assessment of human errors, a number of researchers have improved upon the defects in these systems. For example, Julius [18] proposed the procedure response matrix approach (PRMA), which involves placing faults on one axis of the matrix and symptoms on the other axis to identify commission errors. Guo and Sun [19] constructed the improved analytic hierarchy process HEART method to assess the human reliability of different flight phases and identify the pivotal PSF. Kennedy and Kirwan [20] proposed the Human Hazard and Operability Analysis (Human HAZOP), which is a systematic hazard identification method for predicting potential adverse consequences in the human implementation of the operational process. Yu et al. [21] introduced human error criticality analysis (HECA) as a means to recognize potentially critical human errors during human implementation. Zhang [22] introduced the human error mode effect and criticality analysis (HEMECA) method to determine the ranking of error modes and reduce human errors. This model was built based on the failure mode effects and criticality analysis (FMECA) used to assess hardware equipment. Deacon et al. [23] used the accident risk assessment method for industries (ARAMIS) to select safety barriers and prevent the risk of human error. However, these methods fail to provide complete definition for human error risk, and therefore, they are difficult to apply directly to human error risk quantification for flight safety assessment.

From the perspective of flight safety, human error risk assessment is the quantification of the risks caused by the combined effects of three indicators, i.e., human error possibility, the impact of human errors on the system, and the consequence. These indicators have to be propositioned through sets of language descriptions. Fuzzy logic is advantageous in expressing qualitative knowledge and experience without imposing boundaries, and it provides a promising tool to work with qualitative terms in evaluating risk factors [24–28]. Based on the aforementioned, this study proposes a fuzzy risk assessment of human error (FRAHE) method for quantifying human error risks and identifying the risk severity of critical error modes, which can clearly define human error risk for flight safety assessment. This method does not only consider human errors and their impact and consequences on the system but the relative weights of the errors as well. The remainder of this study is organized as follows. Section 2 provides a brief description of the fuzzy inference system. Section 3 is focused on the fuzzy risk assessment model of human error. A case study and discussion are presented in Section 4. Finally, Section 5 concludes the work and provides recommendations regarding future work.

## 2. Brief description of the fuzzy logic system

**Fig 1:**
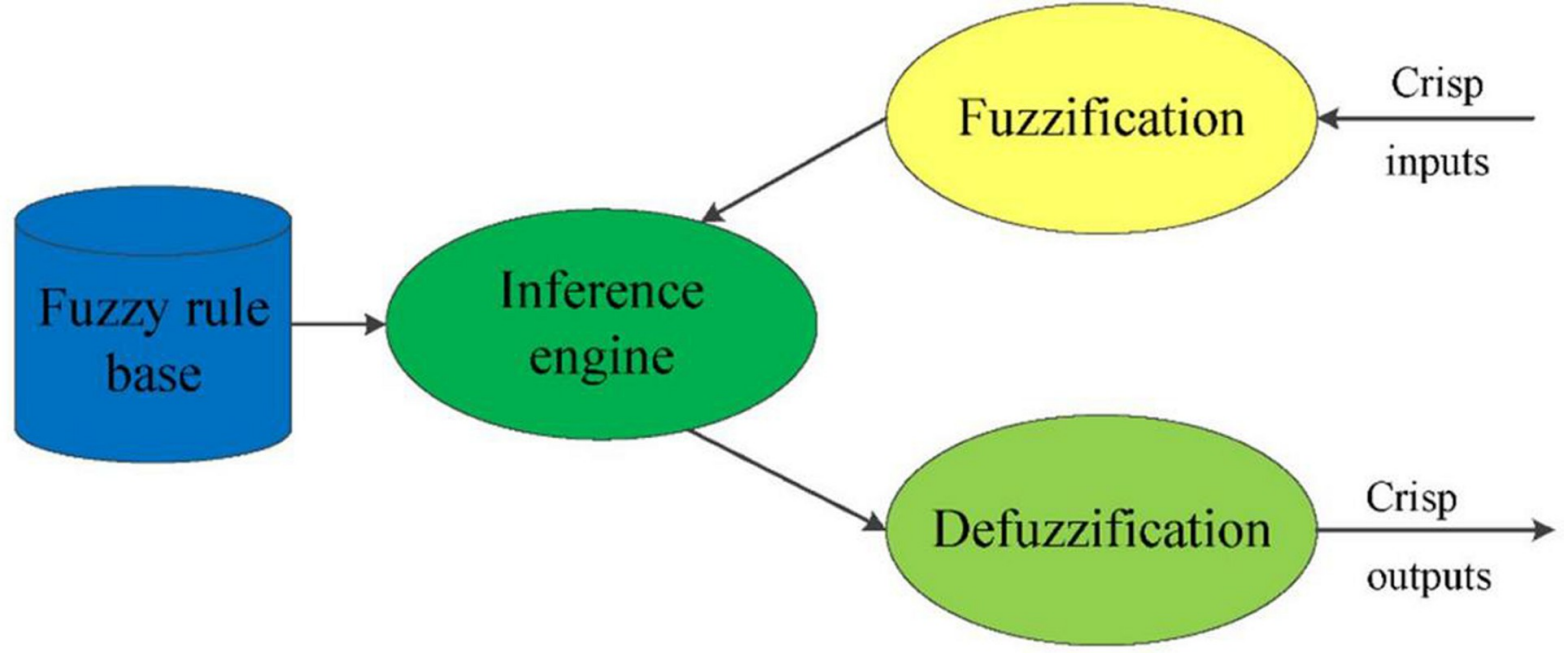
The framework of the fuzzy inference system.

Zadeh [29] first introduced fuzzy logic as a means of representing vagueness and imprecise information. This method begins with the proposal of a fuzzy set, which is a set without a clear boundary. The definition of sets in fuzzy logic is different from that in classical logic. The foundation of classical set theory is binary logic, where any element in the domain is either a member of a set or not. Conversely, fuzzy set theory states that any element in the domain can belong to more than one set. Fuzzy logic is a computing method based on the degree of truth rather than the binary assessment of truth, and it introduces the concept of the membership function [30]. A fuzzy reasoning system generally involves four components: fuzzification, a fuzzy rule base, a fuzzy inference engine, and defuzzification. Fig 1 shows the structure of the fuzzy reasoning system.

### 2.1 Fuzzification

The input signal of the fuzzy inference system consists of real-valued or qualitative variables. However, the fuzzy inference engine can process the fuzzy set signal but cannot process a real-valued signal. The real-valued signal must be fuzzified prior to the fuzzy inference operation. Fuzzification is the process of shifting the crisp value of input variables to one or more fuzzy sets that are completed through use of the information contained in the knowledge base.

### 2.2 Fuzzy rule base

A fuzzy rule base is the core of the fuzzy logic system. It consists of a series of fuzzy rules in IF-THEN form, which describes the local input‒output relationship of the system. The fuzzy IF-THEN rules have the following structure [31]:

$$\begin{array}{c} R^{\left(l\right)}:\lambda_{i}\left(\text{IF}\;x_{1}\;\text{is}\;A_{1}^{l}\;\text{and}\;x_{2}\;\text{is}\;A_{2}^{l}\;\text{and}\;\cdot \cdot \;\text{and}\;x_{n}\;\text{is}\;A_{n}^{l},\;\text{THEN}\;y=B^{l}\right),i=1,2,\ldots ,n,\\ l=1,2,\ldots ,m. \end{array}$$ (1)

where *R*^*l*^ is the *l*-th rule of the fuzzy rule and *x* = (*x*_1_, *x*_2_, …, *x*_*n*_)^*T*^ ∈ *U* and *y* ∈ *V* are the input and output linguistic variables, respectively. $A_{i}^{l}$ and *B*^*l*^ are the fuzzy sets of input and output variables, respectively; *λ_i_* is the rule weight; and $\sum_{i=1}^{n}\lambda_{i}=1$.

### 2.3 Fuzzy inference engine

In the fuzzy inference engine, the fuzzy sets *A* of the input linguistic variables *U* are mapped to the fuzzy sets *B* of the output linguistic variables *V* through the fuzzy IF-THEN rules. Then, the output *B* can be expressed as [32]:

$$\mu_{B^{l}}(y)=\overset{m}{\underset{l=1}{\max}}\left[\sup\;\min(\mu_{A^{l}}(x),\mu_{A_{1}^{l}}(x_{1}),\ldots ,\mu_{A_{n}^{l}}(x_{n}),\mu_{B^{l}}(y))\right]$$ (2)

**Fig 2:**
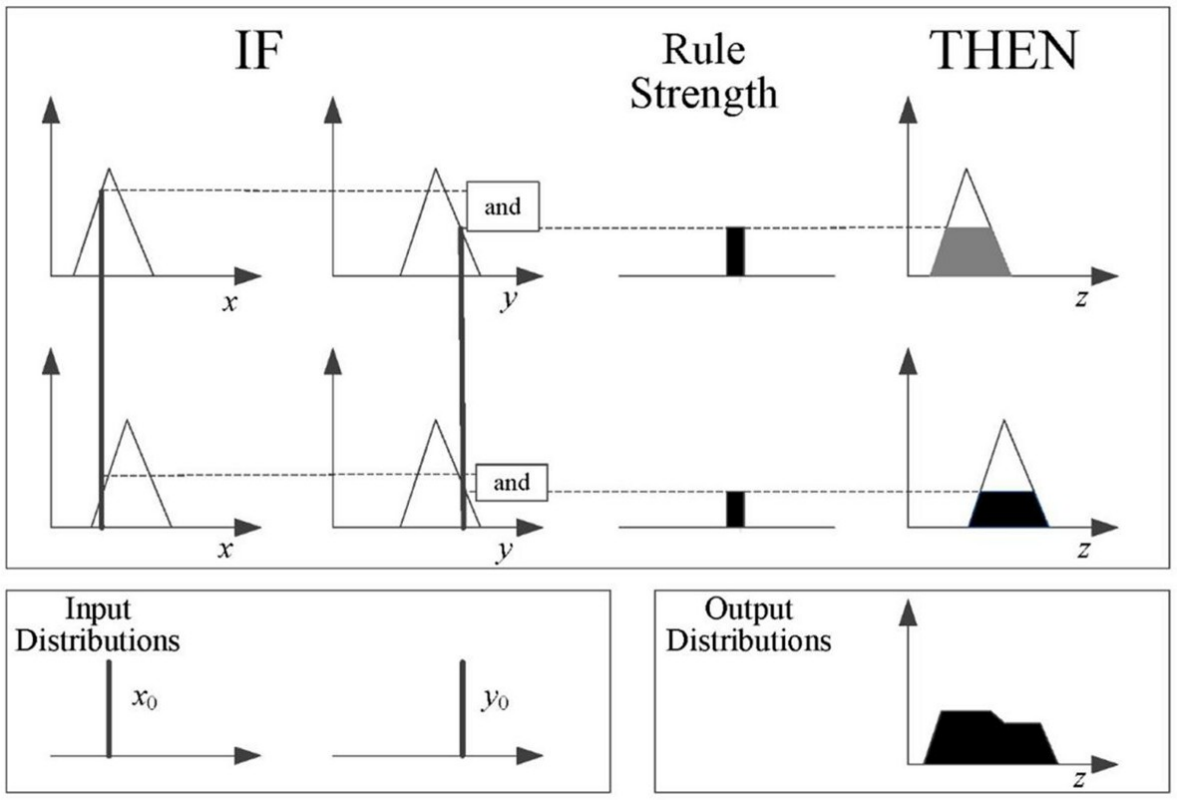
The MFIS block diagram.

Numerous methods for obtaining fuzzy inference systems have been proposed and successfully applied in various industries. In this paper, a Mamdani fuzzy inference system (MFIS) is constructed using the mix-max fuzzy inference method to construct a human error risk model. The MFIS principle with two inputs and one output is illustrated in Fig 2 [32].

### 2.4 Defuzzification

Defuzzification is the process of determining a quantitative result via fuzzy rules and corresponding membership degrees. The fuzzy set *B*^*l*^ of the output signal *V* is eventually mapped to a crisp and defuzzy value. There are various approaches for defuzzification, such as adaptive integration, the centroid of area, and fuzzy clustering defuzzification [33]. This paper uses the centroid of area method to reflect the information represented by the fuzzy set *B*^*l*^. It is expressed as:

$$z^{*}=\frac{\int_{z}z\mu_{B}(z)dz}{\int \mu_{B}(z)dz},$$ (3)

where z^*^ is the defuzzified value, and *μ_B_*(*z*) is the aggregated membership function.

## 3. Fuzzy risk assessment of human error

**Fig 3:**
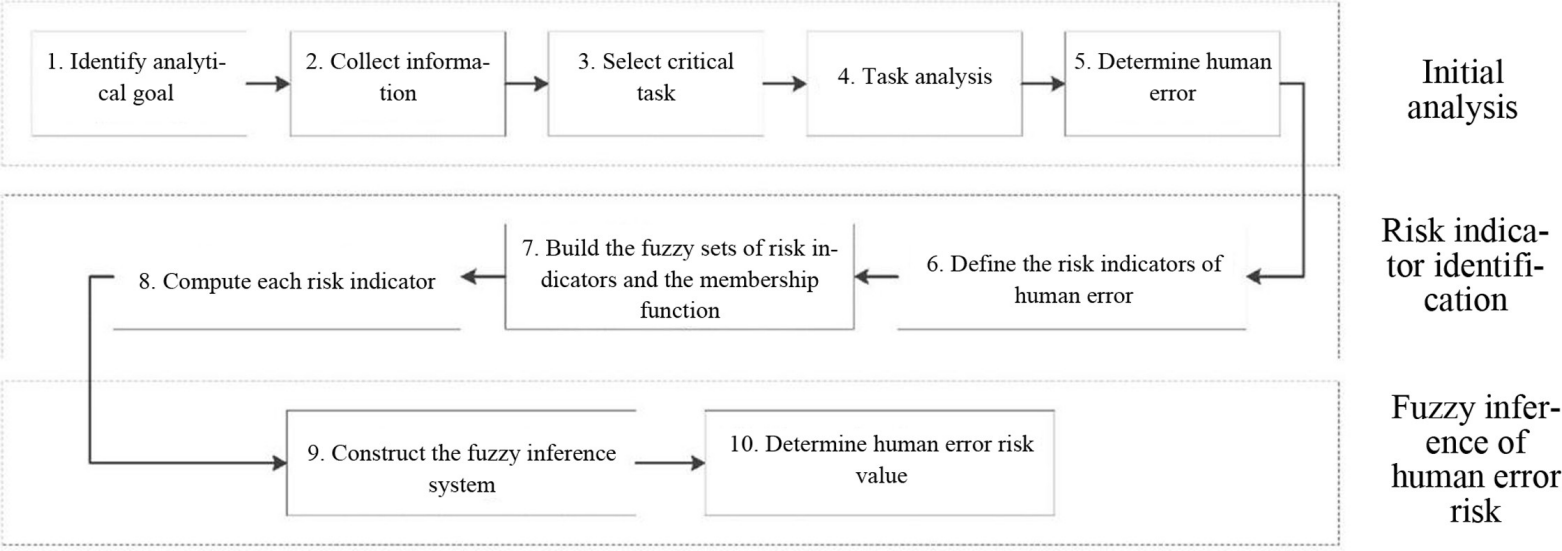
The framework of the risk quantitative model of human error.

To effectively solve the uncertainty and ambiguity of human error risk identification that arises from insufficient information and data, we propose a fuzzy risk assessment model of human error in this paper. Fig 3 shows the general framework of the proposed model, which involves three main stages. i) Preliminary analysis, ii) the identification of human error risk indicators, and iii) the risk assessment of human error.

### 3.1 Initial analysis

In the preliminary analysis stage, there are five parts, namely, specific analytical object identification, information collection, key task selection, task analysis and determination of probable human error. To complete a flight mission, an aircraft must go through several stages, i.e., taxiing, takeoff, climb, cruise, descent, approach, and landing. Since approach is one of the stages with the highest accident rate as well as the most dangerous in an entire flight [34], we select tit as the valuable analysis object first. The information as to the approach stage and cockpit situational environment are collected from the B737-800 flight crew operations manual and Aviation Safety Reporting System and then analyzed, with the key task to screen the tasks that flight crew participate in during the approach stage. Hierarchical task analysis (HTA) is applied to exhaustively decomposes the target task into a series of subtask units until these units cannot be further broken down. Finally, the human error risks are determined using the FRAHE method proposed in this study.

### 3.2 Input parameter determination for the FRAHE model

#### 3.2.1 Human error risk definition

Human error risk assessment is the quantification of the risks caused by the combined effects of three indicators, i.e., human error possibility, the impact of human error on the system, and the consequences. The three indicators are the input variables of the proposed FRAHE method, and the output is risk severity (RS). Assuming that the weights of the three risk indicators are not considered, the quantitative risk value of human error can be expressed as:

$$RS_{FRAHE}=\alpha \times \beta \times \varphi ,$$ (4)

where *α* is human error probability (HEP); *β* is human error impact probability (EIP), which refers to the conditional probability that the human error influences the identified severity classification given the foregone human error mode; and *φ* is human error consequence (HEC).

#### 3.2.2 Fuzzy sets of risk indicators

According to the CREAM approach [15] and expert discussion, HEPs can be classified into five probability intervals, as listed in Table 1, which are depicted in the qualitative term set as follows:

HEP={Verylow,Low,Moderate,High,Veryhigh},

**Table 1:** The risk indicators and corresponding qualitative terms.

Risk indicators	Qualitative term	Value range
HEP	Very low (V-L)	5.0E-6 ≤ *α ≤* 1.0E-3
	Low (L)	1.0E-4 ≤ *α ≤* 1.0E-2
	Moderate (M)	1.0E-3 ≤ *α ≤* 1.0E-1
	High (H)	1.0E-2 ≤ *α ≤* 5.0E-1
	Very High (V-H)	1.0E-1 ≤ *α ≤* 1
EIP	Almost no effect (A-N-E)	0 ≤ *β* < 5.0E-2
	Possible effect (Po-E)	0 ≤ *β* < 5.5E-1
	Probable effect (Pr-E)	5.0E-2 < *β* < 1
	Absolute effect (A-E)	5.5E-1 < *φ* ≤ 1
HEC	Very low (V-L)	0 ≤ *φ* < 2.5E-1
	Low (L)	0 < *φ* < 5.0E-1
	Moderate (M)	2.5E-1 < *φ* < 7.5E-1
	High (H)	5.0E-1 < *φ* < 1
	Very High (V-H)	7.5E-1 < *φ* ≤ 1

**Fig 4:**
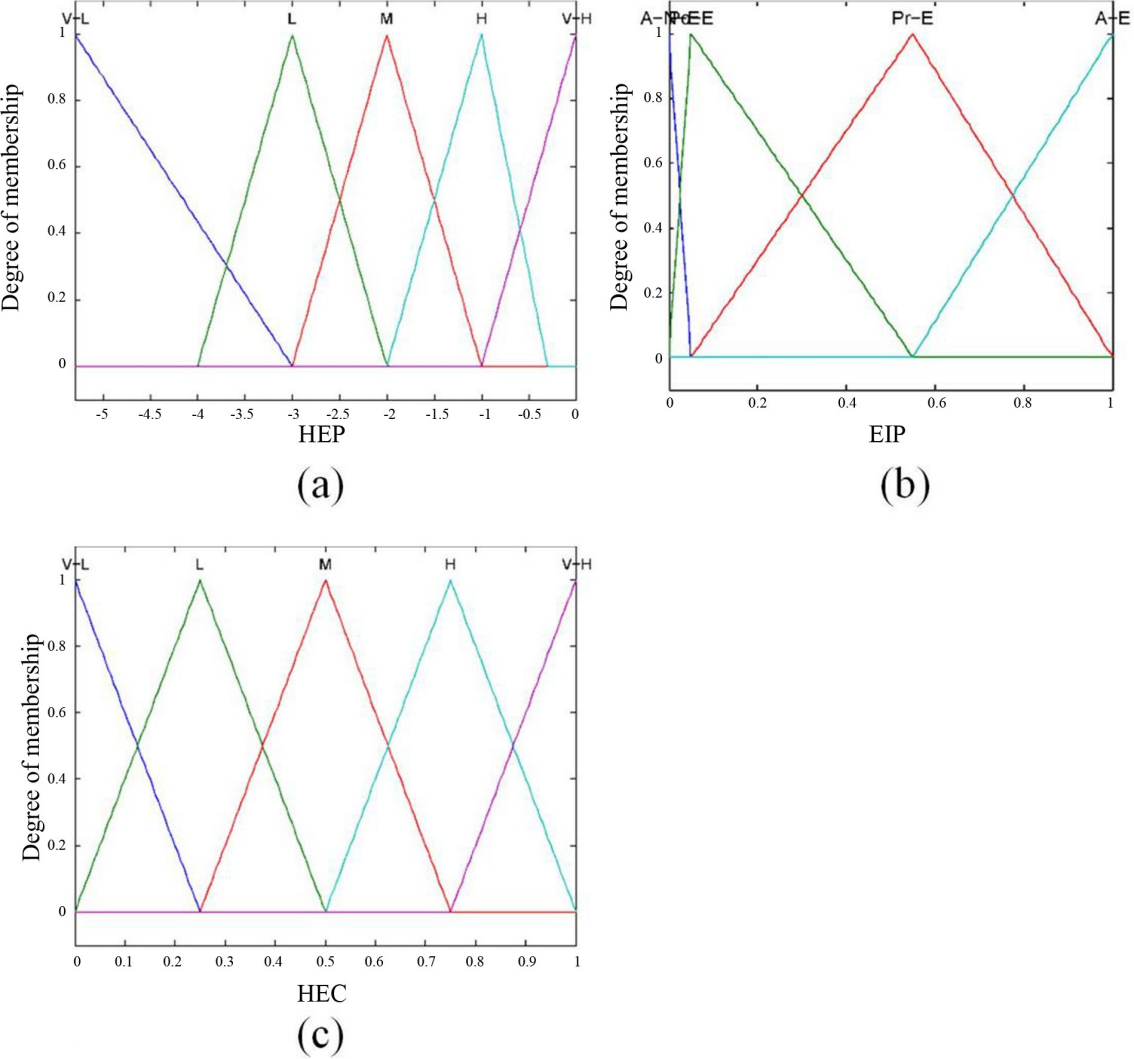
The membership function of the risk indicators.

The five attributes of human error probability are treated as fuzzy sets. The membership function is a mathematical tool for representing fuzzy sets. A membership between 0 and 1 is used to denote the degree to which a crisp HEP value belongs to the fuzzy sets. In this paper, we assume that the membership function of each fuzzy set is the triangular membership function. Fig 4(A) shows the HEP fuzzy sets and corresponding membership functions. We use the logarithm of the HEP as the x-axis for better output representation.

According to MIL-STD-1629A, the EIP fuzzy sets are similarly allocated to four qualitative levels. For the severity classification of HECs, we classify the consequences of human error into five qualitative levels in light of the loss percentage of the system. The EIP and HEC are described in linguistic terms as follows:

EIP={Almostnoeffect,Possibleeffect,Probableeffect,Absoluteeffect},

HEC={Verylow,Low,Moderate,High,Veryhigh}

The fuzzy sets and membership functions of the EIP and HEC are presented in Fig 4(B) and 4(C), respectively.

#### 3.2.3 Calculation of the risk indicators

Numerous human error quantitative approaches, such as THERP, SLIM, HEART, and CREAM, have been proposed for calculating the probability of human error and have been successfully applied in various industries. In this paper, a simplified CREAM approach is employed to determine the HEP of each task unit. This approach not only accounts for task features but also the effect of context on human cognition and action. The probability of human error can be expressed as [35]:

$$HEP=CFP_{0}\times 10^{0.25\sum_{i=1}^{n}\rho_{i}}$$ (5)

where *CFP*_0_ is the nominal probability of human cognitive failure. *ρ_i_* is the *i*-th performance influence index, which represents the effect of the *i*-th task context factor on human error.

The defuzzification method based on fuzzy theory is used to determine the crispness values of the EIP and HEC. Generally, based on their knowledge and experience, engineers or analysts are needed for measuring these risk indicators. Such professionals can provide a precise value, a numerical range, a qualitative term, or a triangular fuzzy value. If the information is sufficient and the indicator is a quantitative metric, experts or engineers can offer a precise value via the defuzzification method. However, under insufficient and uncertain information, it is not easy for engineers to provide an exact value. A qualitative level or a fuzzy value may be more appropriate in such a context. Then, we use the defuzzification method of the triangular center of gravity to obtain the crisp value, which is expressed as [36]:

$$z_{i}^{*}=\frac{\left(a_{i}-c_{i})+(b_{i}-c_{i}\right)}{3}+c_{i},$$ (6)

where $z_{i}^{*}$ is an exact value converted from the triangular membership function. *a*_*i*_ and *c*_*i*_ are the ‘feet’ of the triangle, and *b*_*i*_ is the ‘peak’ of the triangle. For example, if the EIP is assessed as a probable effect, and the corresponding triangular fuzzy number is (0.5, 0.4, 0.6), then the crisp value is 0.5, as calculated using Eq 6.

### 3.3 Fuzzy inference output of human error risk

#### 3.3.1 Establishment of the fuzzy rule base

Before constructing the fuzzy rule base, we need to assign the output variable risk severity to ten linguistic levels on the basis of expert opinion and reference. The risk severity is depicted with the qualitative term and numerical intervals as follows [37]:

$$\begin{array}{l} RS=\{Unnecessary,\;Minor,\;Very\;low,\;Low,\;Moderate,\;Slightly\;high,\\ \;Moderate\;high,\;Very\;high,\;Extremely\;high,\;Absolutely\;high\}\\ \;=\{\left[0,0.2\right],\left[0.1,0.3\right],\left[0.2,0.4\right],\left[0.3,0.5\right],\left[0.4,0.6\right],\left[0.5,0.7\right],\\ \;\left[0.6,0.8\right],\left[0.7,0.9\right],\left[0.8,1.0\right],\left[0.9,1.0\right],\} \end{array}$$

**Fig 5:**
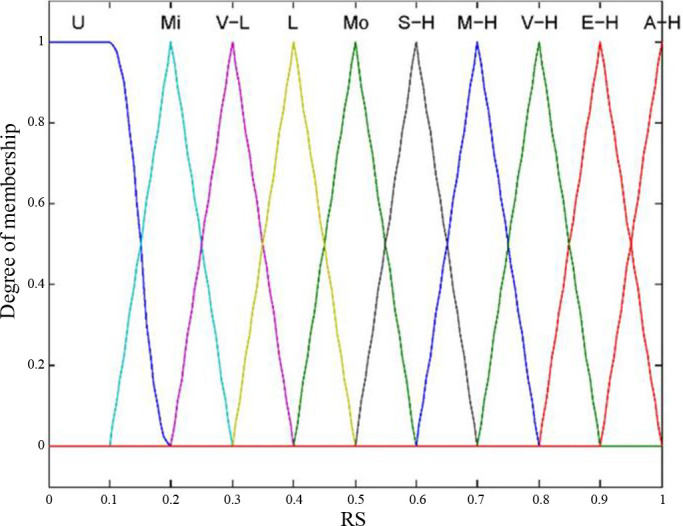
Represents the fuzzy sets and the membership function of the RS.

The fuzzy sets and membership function of the RS are shown in Fig 5.

Then, the fuzzy rule base is generated by the IF-THEN rule as informed by expert discussion and relevant information based on previous human error analysis. In this section, we integrate the relative weights of the risk indicators into the fuzzy set of each input variable. The relative weights of the HEP, EIP, and HEC are 0.4, 0.2, and 0.4, respectively, according to expert judgment. The logical AND operator is utilized as the constructing mode. The Fuzzy Logic Toolbox in MATLAB is used to construct a fuzzy inference system. There are 100 (5x4x5) rules addressed in this paper, and some of these rules are listed as follows:

Rule 1: if (HEP is V-L) and (EIP is A-N-E) and (HEC is V-L), then (RS is U)
Rule 2: if (HEP is V-L) and (EIP is Po-E) and (HEC is V-L), then (RS is U)
Rule 3: if (HEP is L) and (EIP is A-N-E) and (HEC is L), then (RS is Mi)
Rule 11: if (HEP is L) and (EIP is Po-E) and (HEC is V-L), then (RS is Mi)
Rule 14: if (HEP is L) and (EIP is Po-E) and (HEC is L), then (RS is V-L)
Rule 17: if (HEP is L) and (EIP is Pr-E) and (HEC is V-L), then (RS is V-L)
Rule 21: if (HEP is L) and (EIP is A-E) and (HEC is V-L), then (RS is L)
Rule 23: if (HEP is M) and (EIP is A-N-E) and (HEC is M), then (RS is L)
Rule 35: if (HEP is L) and (EIP is Po-E) and (HEC is M), then (RS is Mo)
Rule 38: if (HEP is L) and (EIP is Pr-E) and (HEC is M), then (RS is Mo)
Rule 50: if (HEP is V-L) and (EIP is Po-E) and (HEC is V-H), then (RS is M-H)
Rule 55: if (HEP is M), (EIP is A-E) or (HEC is L), then (RS is M-H)
Rule 62: if (HEP is L) and (EIP is Po-E) and (HEC is V-H), then (RS is H)
Rule 66: if (HEP is V-H), (EIP is A-E) or (HEC is M), then (RS is H)
Rule 71: if (HEP is H) and (EIP is Po-E) and (HEC is H), then (RS is V-H)
Rule 75: if (HEP is H) and (EIP is Pr-E) and (HEC is H), then (RS is V-H)
Rule 83: if (HEP is H) and (EIP is A-E) and (HEC is V-H), then (RS is N)
Rule 88: if (HEP is V-H) and (EIP is A-N-E) and (HEC is V-H), then (RS is N)

**Fig 6:**
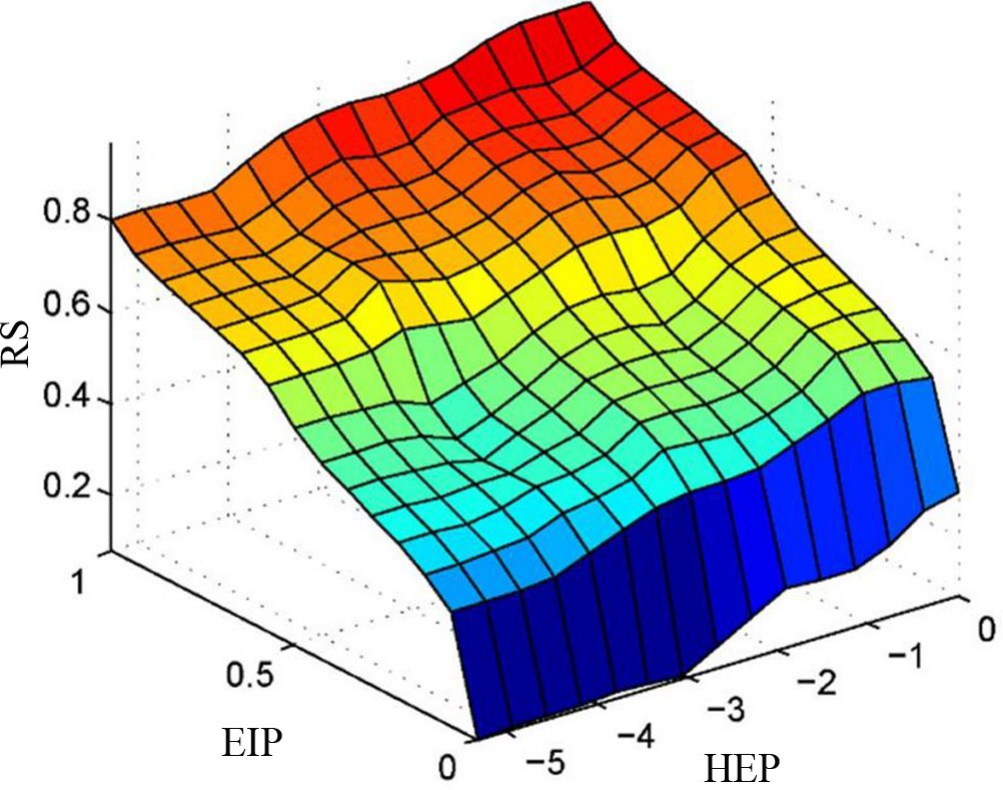
Fuzzy mapping of two inputs to a single output.

**Fig 7:**
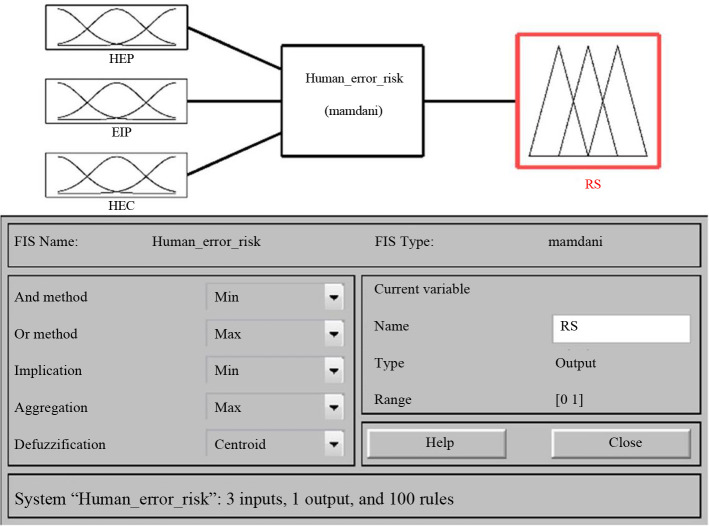
Fuzzy inference system for human error risk.

These fuzzy rules are the core of the fuzzy inference system, which can transform the input variables into the output variables. The fuzzy mapping of two inputs to a single output is presented in Fig 6. Fig 7 shows the fuzzy inference system of human error risk.

#### 3.3.2 Determination of the severity of human error risk

The experts or analysts are needed to check the final output to ensure that the decision is reliable. When several illogical outputs are found, they should be corrected in a timely manner. For example, when the system structure changes, the influence of the risk indicators is not fully estimated. In this context, the expert group should collect more information associated with the task under analysis to reassess the quantitative risk process and adjust the values of risk indicators. Eventually, the risk severity of human error is calculated and ranked according to the output results. The results may provide technical guidance for flight safety management and further guide appropriate risk decision-making.

## 4. Case study

### 4.1 Initial analysis

According to historical data, numerous serious unsafe incidents in civil aviation occurred during nonprecision approaches [38]. A nonprecision approach refers to a kind of instrument approach that uses a navigation system for course deviation but does not offer a glide-path guide. This type of flight is characterized by a lack of instrument information for directly determining the vertical path. The level of automation is lower than that of the precision approach. In addition, the workload of operators in a nonprecision approach is also greater, which results in an insecure approach operation and controlled flight into terrain (CFIT) (almost 60% of CFIT accidents occur during a nonprecision approach) [39]. Compared with that of a precision approach, the accuracy of a nonprecision approach is much lower, and there are also many restrictions due to unfriendly environments, such as low visibility, low clouds, and wind shear. Therefore, human errors are more likely to occur during a nonprecision approach phase than during a precision approach phase. Moreover, according to aviation accident investigations, the accident rate related to human actions during the approach process is obviously greater than that during other flight phases [2]. Eventually, the nonprecision approach under low visibility is selected to demonstrate the proposed risk assessment method for human error.

After collecting valuable information and determining the analysis object, we use the HTA method to decompose the approach procedures into a sequence of operation units. The results are listed in Table 2. The potential human error type and nominal value, as shown in Table 3, can be identified through the CREAM method, which provides an exhaustive description of the identified human errors [15]. Next, operation unit 1.1 is taken as an example to show the calculation process for human error risk.

**Table 2:** A sequence of operation units based on the HTA.

Task	The description of sequential operation
1. Initial approach	1.1 Set the LANDING light switches to ON at or above 10000 ft
	1.2 Set and crosscheck the altimeters at the transition level
	1.3 Update the arrival and approach procedures as needed
	1.4 Update the navigation performance as needed
	1.5 Update the approach briefing as needed
	1.6 Call and perform the "APPROACH CHECKLIST"
2. Final approach	2.1 Call and set the flap lever
	2.2 Monitor the flap and slat extension
	2.3 Verify the instrument landing system and identify when on the localizer intercept heading
	2.4 Verify that LOCALIZER and GLIDE SLOPE are shown when on the localizer intercept heading
	2.5 Use HDG SEL to intercept the final approach course as needed
	2.6 Verify that the localizer is captured
	2.7 Call "GLIDE SLOPE ALIVE"
	2.8 Call "GEAR DOWN" and "FLAP 15"
	2.9 Set the landing gear lever to DN
	2.10 Verify that the green landing gear indicator lights are illuminated
	2.11 Set the flap lever to 15
	2.12 Set the engine start switches to CONT
	2.13 Set the speed brake lever to ARM
	2.14 Verify that the SPEED BRAKE ARMED light is illuminated
	2.15 Call and set the flap lever at glide slope capture
	2.16 Set the missed approach altitude on the MCP
	2.17 Call and perform the "LANDING CHECKLIST"
	2.18 Verify the crossing altitude at final approach
	2.19 Monitor the approach
	2.20 Verify callouts and the autoland status at 500 ft

**Table 3:** Human error types and nominal values.

Cognitive function	Generic failure type	Nominal value
Observation	O1. Wrong object observed	1.0E-3
	O2. Wrong identification	7.0E-2
	O3. Observation not made	7.0E-2
Interpretation	I1. Faulty diagnosis	2.0E-1
	I2. Decision error	1.0E-2
	I3. Delayed interpretation	1.0E-2
Planning	P1. Priority error	1.0E-2
	P2. Inadequate plan	1.0E-2
Execution	E1. Action of a wrong type	3.0E-3
	E2. Action at wrong time	3.0E-3
	E3. Action on wrong object	5.0E-4
	E4. Action out of sequence	3.0E-3
	E5. Missed action	3.0E-2

### 4.2 Calculation of the input parameters of the FRAHE model

#### 4.2.1 Human error probability

The simplified CREAM algorithm is used to compute the HEP of each operation unit. First, the task context is defined on the basis of expert experience and knowledge. The context factors affecting human performance during the approach stage mainly involved 9 categories: ground support, crew work management, training and experience, procedure quality, procedure quantity, time stress, the adequacy of the human‒machine interface, and the adequacy of the organization and work conditions. The level of the performance influence factors and the corresponding value of the performance influence index are determined based on the CREAM and expert discussions [35]. The results are listed in Table 4. Then, we can determine the human error probability of each operation unit with Eq (5). For instance, the cognitive activity is ‘execute’ in operation unit 1.1. The corresponding cognitive function is ‘execution’, and the potential failure type is ‘E5’, which indicates that its nominal error probability (*CFP*_0_) is 5.0E-4. Thus, the HEP of operation unit 1.1 in a special context can be calculated with Eq (5) as follows:

$$HEP=CFP_{0}\times 10^{0.25\sum_{i=1}^{n}\rho_{i}}=0.03\times 10^{0.25\times (-2.4)}=7.5\text{E}-3.$$

**Table 4:** The task context in approach.

Performance influence factors	PSF Level	Performance influence index
Ground support	Efficient	0
Crew workload management	Adequate	-1.0
Crew training and experience	High experience	-1.8
Quality of procedures	Appropriate	-1.6
Quantity of procedures	Matching current capacity	0
Time stress	Temporarily inadequate	1.0
Adequacy of the man-machine interface	Adequate	-0.6
Adequacy of organization	Efficient	0
Working conditions	Incompatible	1.6

To facilitate the calculation of human error risk, this study takes the logarithm of the HEP in operation unit 1.1, and the result is -2.12. Similarly, the HEP of other operation units are determined separately, as shown in Table 5.

**Table 5:** Results of risk assessment for human errors.

Task	Operation unit	Cognitive activity	Potential error type	CFP0	HEP	Error impact	EIP	HEC	RS
1. Initial approach	1.1	Execute	E5	3.0E-2	7.53E-3 (-2.12)	Reflected in process	Po-E (0.5)	L (0.333)	0.638
	1.2	Coordinate	E5	3.0E-2	7.53E-3 (-2.12)	Set wrong procedure	1	V-H (0.92)	0.9
	1.3	Coordinate	E1	3.0E-3	7.53E-4 (-3.12)	Reflected in process	Po-E (0.5)	M (0.513)	0.554
	1.4	Coordinate	E1	3.0E-3	7.53E-4 (-3.12)	Reflected in process	Po-E (0.5)	M (0.513)	0.554
	1.5	Evaluate	E1	3.0E-3	7.53E-4 (-3.12)	Reflected in process	Po-E (0.5)	M (0.513)	0.554
	1.6	Coordinate	E2	3.0E-3	7.53E-4 (-3.12)	Reflected in process	Pr-E (0.7)	H (0.73)	0.717
2. Final approach	2.1	Coordinate	E5	3.0E-2	7.53E-3 (-2.12)	Set wrong procedure	1	V-H (0.92)	0.9
	2.2	Monitor	O2	7.0E-2	1.76E-2 (-1.75)	Latent failure occurred	Po-E (0.5)	L (0.333)	0.641
	2.3	Verify	O2	7.0E-2	1.76E-2 (-1.75)	Reflected in process	Pr-E (0.7)	M (0.513)	0.783
	2.4	Verify	O2	7.0E-2	1.76E-2 (-1.75)	Reflected in process	Pr-E (0.7)	M (0.513)	0.783
	2.5	Evaluate	E1	3.0E-3	7.53E-4 (-3.12)	Set wrong procedure	1	V-H (0.92)	0.892
	2.6	Verify	O3	7.0E-2	1.76E-2 (-1.75)	Reflected in process	Pr-E (0.7)	L (0.333)	0.783
	2.7	Communicate	E4	3.0E-3	7.53E-4 (-3.12)	Reflected in process	Po-E (0.5)	L (0.333)	0.552
	2.8	Communicate	E4	3.0E-3	7.53E-4 (-3.12)	Reflected in process	Po-E (0.5)	H (0.73)	0.554
	2.9	Execute	E5	3.0E-2	7.53E-3 (-2.12)	Set wrong procedure	1	V-H (0.92)	0.9
	2.10	Verify	O2	7.0E-2	1.76E-2 (-1.75)	Latent failure occurred	Pr-E (0.7)	L (0.333)	0.783
	2.11	Execute	E5	3.0E-2	7.53E-3 (-2.12)	Set wrong procedure	1	V-H (0.92)	0.9
	2.12	Execute	E5	3.0E-2	7.53E-3 (-2.12)	Reflected in process	1	V-H (0.92)	0.9
	2.13	Execute	E5	3.0E-2	7.53E-3 (-2.12)	Set wrong procedure	1	V-H (0.92)	0.9
	2.14	Verify	O2	7.0E-2	1.76E-2 (-1.75)	Reflected in process	Po-E (0.5)	H (0.73)	0.641
	2.15	Coordinate	E5	3.0E-2	7.53E-3 (-2.12)	Reflected in process	Pr-E (0.7)	V-H (0.92)	0.76
	2.16	Execute	E5	3.0E-2	7.53E-3 (-2.12)	Not set in time	1	V-H (0.92)	0.9
	2.17	Coordinate	E4	3.0E-3	7.53E-4 (-3.12)	Reflected in process	Pr-E (0.7)	H (0.73)	0.717
	2.18	Verify	I2	1.0E-2	2.51E-3 (-2.60)	Reflected in process	Pr-E (0.7)	H (0.73)	0.722
	2.19	Monitor	I2	1.0E-2	2.51E-3 (-2.60)	Reflected in process	Po-E (0.5)	M (0.513)	0.61
	2.20	Verify	O2	7.0E-2	1.76E-2 (-1.75)	Latent failure occurred	Po-E (0.5)	M (0.513)	0.61

#### 4.2.2 Error impact probability

EIP is the conditional probability, which is the probability of the influence of an error on the identified consequence classification given the specific human error. If a human error occurs in the *i*-th operation unit, this error can result in a certain level of system loss with any truth. The truth level of the identified consequence classification is the EIP, and the value range of the truth level is between 0 and 1. In this section, the case of operation unit 1.1 is considered. The potential human error type is ‘E5’, which results in a low degree of system loss. The truth level is determined by expert judgment using the triangular fuzzy number (0.5, 0.6, 0.4). Eventually, the crisp value of the EIP is calculated through Eq (6), and the result is 0.5. This finding implies that the impact of operation unit 1.1 error on the system is ‘Possible effect’. Similarly, other diagnostic results are presented in Table 5.

#### 4.2.3 Human error consequences

Human error can affect mechanical system, avionics system, system function, the environment, and even human safety. The metric of human error consequence can include reliability, availability, safety, cost, etc. In this study, we categorize the human error consequence based on the cost-loss proportion of the system, as listed in Table 1. It is assumed that the effect of cognitive errors lags and may be reflected in the subsequent cognitive process. For instance, observation errors must influence operating errors. In this section, we similarly use the triangular fuzzy number to determine the crisp value of the HEC. The triangular fuzzy number of operation unit 1.1 follows (0.2, 0.4, 0.4) according to expert knowledge and experience. Consequently, the corresponding measurement result is 0.333, as calculated by Eq (6). Table 5 shows all the analytical results of the HEC.

### 4.3 Output of fuzzy inference for human error risk

**Fig 8:**
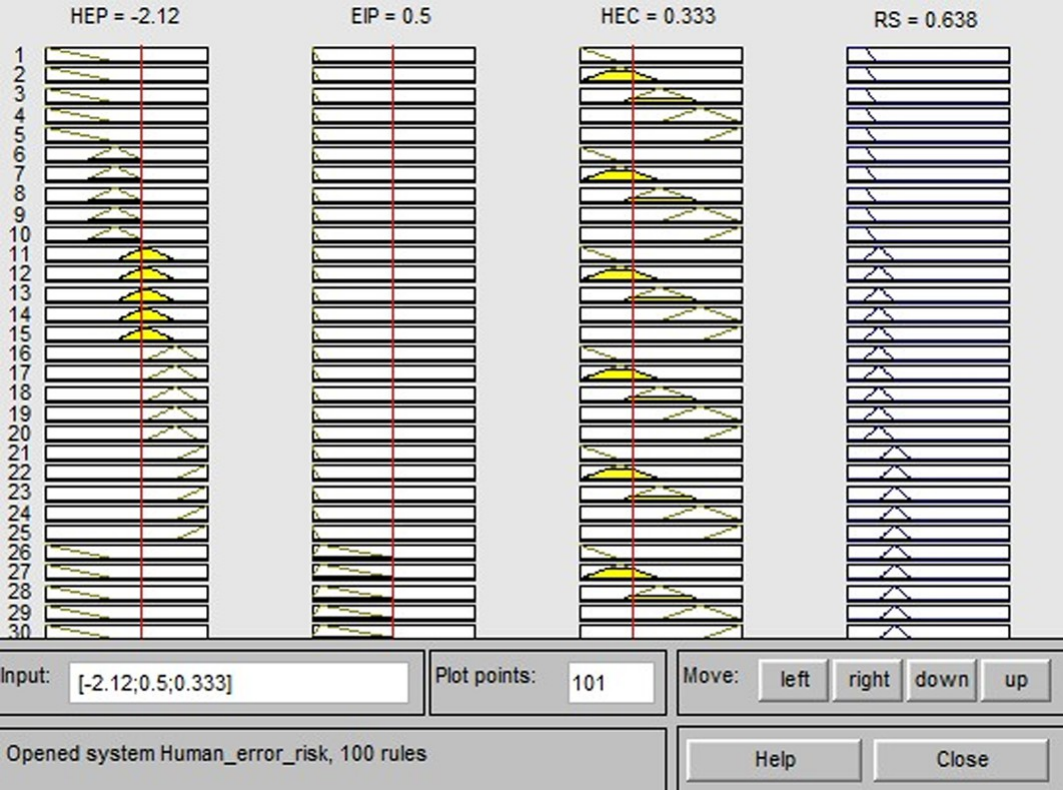
Fuzzy inference process of human error risk.

Take operation unit 1.1 as an example. The HEP value is 7.53E-3 (its logarithm is -2.12), computed by the simplified CREAM method. The EIP and HEC are calculated using the defuzzification method of the triangular center of gravity. The EIP result is 0.5, which is within the value range of (0, 0.55). This indicates that the impact of human error in operation unit 1.1 on the system is ‘possible effect (Po-e)’. The HEC result is 0.333 and lies within the interval of (0, 0.5), which suggests that the system loss caused by human error in unit 1.1 is ‘low’. After the three risk indicators (HEP, EIP and HEC) are calculated, they are separately input into the proposed FRAHE model through the Fuzzy Logic Toolbox in MATLAB. The fuzzy inference process of unit 1.1 is presented in Fig 8, and the RS value is 0.638. The RS values for all other operation units are determined in a similar manner, as shown in Table 5. The results indicate that the most critical cognitive error type is ‘E5’ (i.e., missed action), which is found in operation unit 1.2, unit 2.1, unit 2.9, unit 2.11, unit 2.12, unit 2.13, and unit 2.16. Their risk values are 0.9. The main reason for this is that operators are prone to overlooking certain actions while performing extensive tasks in a short duration. If these missed actions occur, the consequences are very serious. Thus, airlines should pay more attention to improving these operation units. The second critical error type is ‘E1’ (i.e., action of wrong type), as seen in operation unit 2.5, and its risk value is 0.892. The conducting of diagnosis under these conditions is an experience-based action, and the probability of diagnosis error is high. The error type ‘O2’ (i.e., wrong identification) seen in unit 2.3, unit 2.4, and unit 2.10 is also a critical human error. The risk values of the other error types can be found in Table 5. The risk severity of each operation unit can be identified based on these risk values. Moreover, airlines can establish proper rules and management measures to prevent human error occurrence according to the risk prioritization (RP) of these operations.

### 4.4 Comparison of FRAHE with other methods

To demonstrate the availability and reasonability of the proposed method, the results of this method are compared with those of other methods. The risk prioritization of human error with different methods is listed in Table 6. The HEP is directly utilized to evaluate human error risk in the simplified CREAM approach. The original HECA method employs the product of HEP, EIP, and HEC to determine the critical error types and the risk severity of human errors. In this paper, we develop a fuzzy risk assessment of human error method, which uses the above three variables as risk indicators and applies the fuzzy logic system to identify the risk prioritization of human errors.

**Table 6:** Comparison of method results.

Operation unit	Potential error type	HEP	EIP	HEC	HECA	FRAHE	RP with CREAM	RP with HECA	RP with FRAHE
1.1	E5	7.53E-3 (-2.12)	Po-E (0.5)	L (0.333)	1.17E-3	0.64	2	9	8
1.2	E5	7.53E-3 (-2.12)	1	V-H (0.92)	6.93E-3	0.9	2	1	1
1.3	E1	7.53E-4 (-3.12)	Po-E (0.5)	M (0.513)	1.93E-4	0.554	4	14	10
1.4	E1	7.53E-4 (-3.12)	Po-E (0.5)	M (0.513)	1.93E-4	0.554	4	14	10
1.5	E1	7.53E-4 (-3.12)	Po-E (0.5)	M (0.513)	1.93E-4	0.554	4	14	10
1.6	E2	7.53E-4 (-3.12)	Pr-E (0.7)	H (0.73)	3.85E-4	0.717	4	12	6
2.1	E5	7.53E-3 (-2.12)	1	V-H (0.92)	6.93E-3	0.9	2	1	1
2.2	O2	1.76E-2 (-1.75)	Po-E (0.5)	L (0.333)	2.75E-3	0.641	1	7	7
2.3	O2	1.76E-2 (-1.75)	Pr-E (0.7)	M (0.513)	6.32E-3	0.783	1	3	3
2.4	O2	1.76E-2 (-1.75)	Pr-E (0.7)	M (0.513)	6.32E-3	0.783	1	3	3
2.5	E1	7.53E-4 (-3.12)	1	V-H (0.92)	6.93E-4	0.892	4	10	2
2.6	O3	1.76E-2 (-1.75)	Pr-E (0.7)	L (0.333)	3.86E-3	0.783	1	6	3
2.7	E4	7.53E-4 (-3.12)	Po-E (0.5)	L (0.333)	1.18E-4	0.552	4	15	11
2.8	E4	7.53E-4 (-3.12)	Po-E (0.5)	H (0.73)	2.75E-4	0.554	4	13	10
2.9	E5	7.53E-3 (-2.12)	1	V-H (0.92)	6.93E-3	0.9	2	1	1
2.10	O2	1.76E-2 (-1.75)	Pr-E (0.7)	L (0.333)	3.86E-3	0.783	1	6	3
2.11	E5	7.53E-3 (-2.12)	1	V-H (0.92)	6.93E-3	0.9	2	1	1
2.12	E5	7.53E-3 (-2.12)	1	V-H (0.92)	6.93E-3	0.9	2	1	1
2.13	E5	7.53E-3 (-2.12)	1	V-H (0.92)	6.93E-3	0.9	2	1	1
2.14	O2	1.76E-2 (-1.75)	Po-E (0.5)	H (0.73)	6.42E-3	0.641	1	2	7
2.15	E5	7.53E-3 (-2.12)	Pr-E (0.7)	V-H (0.92)	4.85E-3	0.76	2	4	4
2.16	E5	7.53E-3 (-2.12)	1	V-H (0.92)	6.93E-3	0.9	2	1	1
2.17	E4	7.53E-4 (-3.12)	Pr-E (0.7)	H (0.73)	3.85E-4	0.717	4	12	6
2.18	I2	2.51E-3 (-2.60)	Pr-E (0.7)	H (0.73)	1.28E-3	0.722	3	8	5
2.19	I2	2.51E-3 (-2.60)	Po-E (0.5)	M (0.513)	6.44E-4	0.61	3	11	9
2.20	O2	1.76E-2 (-1.75)	Po-E (0.5)	M (0.513)	4.66E-3	0.61	1	5	9

**Fig 9:**
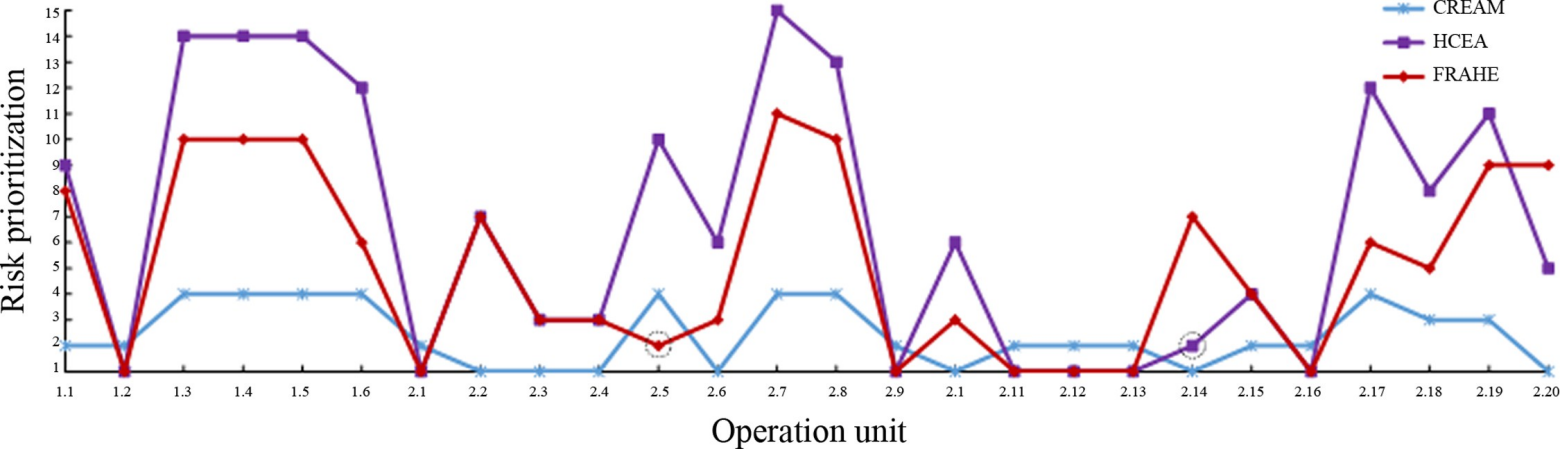
The risk prioritization of human errors under different assessment methods.

To clearly analyze and discuss the differences in human error risk severity in the calculations of these three methods, the assessment results are depicted in Fig 9. In CREAM, the most critical human errors are in operation units 2.2–2.4, unit 2.6, unit 2.10, unit 2.14 and unit 2.20, and the error type is ‘O2’ (i.e., wrong identification). CREAM defines the risk severity of human errors merely based on the HEP. The limitation of CREAM is that it ignores the impacts of human error on the system; thus, this method may not be able to reflect the authentic severity of human error in flight.

In the FRAHE, the most critical risk is observed in operation units 1.2, 2.1, 2.9, 2.11–2.13, and 2.16, similar to that in the HECA. However, their second critical risk points differ: In the FRAHE, the risk priorities for units 2.5 and 2.14 are 2 and 7, whereas in the HECA, the risk priorities for these units are 10 and 2, respectively. Human error in operation unit 2.14 mainly affects the decision to turn on the speed brake light while operation unit 2.5 involves the final landing operation and therefore human error in this unit may cause a catastrophic accident due to missing the best approach fix. Despite a much lower human error probability (Table 6), operation unit 2.5 has a greater risk priority than unit 2.14 in the FRAHE, and therefore, with the FRAHE fits the actual flight situation better than the HECA.

The reason for this disparity between the HECA and the method proposed in this study may be as follows. The risk priority value in the HECA is the simple product of human error probability, error impact probability, and human error consequence, where the three parameters are assumed to be equal in importance. Therefore, it may generate close risk priority values for different combinations of the three input parameters. For example, units 2.6 and 2.20 have the values of (1.76E-2, 0.7, 0.333) and (1.76E-2, 0.5, 0.513) for the three input parameters, respectively, and therefore, the output results based on the HECA for the two units are very comparable. However, the risk significance of human errors in these two operation units can be quite different: It is generally accepted that human error in the former operation is more critical than that in the latter, with the latter involving the influence on aircraft availability due to potential positioning deviation in the system. In contrast, the risk priority values for units 2.6 and 2.20 are 3 and 9 according to the FRAHE, respectively, as a consequence of the integration of expert knowledge and experience, which means that flight crew should take more safety measures for the prioritized unit 2.6. The FRAHE constructs a fuzzy inference model for the risk indicators rather than utilizes a simple product, making it more reasonable and effective than the HECA method.

## 5. Concluding remarks and future work

Human error is one of the most important factors that leads to unsafe events or aviation accidents. In this study, we propose a quantitative risk assessment model based on a fuzzy logic system to identify risk severity of human errors with the expectation to prevent the occurrence of critical errors.

The results of the case analysis of the approach task indicate that “E5: Missed action” is the most critical human error type during the approach stage and that the highest risk severity values among all operating units are 1.2, 2.1, 2.9, 2.11, 2.12, 2.13 and 2.16. These results indicate that to reduce flight human errors for the approach task, airlines or organizational management should strengthen flight crew training for these subtasks and the crew should complete correct operating procedures within specified time. The FRAHE method proposed in this study introduces three risk indicators, i.e., HEP, EIP and HEC, as the input parameters takes risk severity as the output result. It does not only consider human error probability but the effect of human error on the system as well. Furthermore, the integration of the relative weights of the three indicators into the fuzzy rule base improves the uncertainty and effectiveness of risk assessment. Based on the results obtained in this study, the FRAHE method can be used to quantify human error risk for flight safety assessment, and it enables prospective analysis to be performed to prevent unsafe incidents or aviation accidents to a certain extent.

Even though the proposed approach has many advantages, it still has certain limitations. It artificially breaks continuous information in the real world into discrete variables, which matters the issue of fuzzy rules. The prerequisites of this approach include precise value determination for risk indicators and creation of fuzzy rules and membership functions, which are all difficult tasks. Particularly, fuzzy rules and membership functions depend heavily on expert experience and knowledge, which increases the subjectivity and uncertainty of the system. Therefore, larger human error data and the application of artificial intelligence technology in establishing the fuzzy rules and membership functions will be essential to improve the model’s effectiveness in the future.
